# Uncertainty stress and self-rated health during the early stage of the COVID-19 outbreak

**DOI:** 10.1080/21642850.2023.2173202

**Published:** 2023-02-15

**Authors:** Zan Zhu, Dan Wu, Kaigong Wei, Yingbi Liu, Zhe Xu, Guihua Jiao, Lingwei Yu, Alyx Taylor, Liye Zou

**Affiliations:** a Hwa Mei Hospital, University of Chinese Academy of Sciences, Ningbo, Zhejiang, People’s Republic of China; b School of Psychology/ Shenzhen Humanities & Social Sciences Key Research Bases of the Center for Mental Health, Shenzhen University, Shenzhen, Guangdong, People’s Republic of China; c Department of Psychology/Research Center on Quality of Life and Applied Psychology, Guangdong Medical University, Dongguan, Guangdong, People’s Republic of China; d Center for Tobacco Control Research, Zhejiang University School of Medicine, Hangzhou, Zhejiang, People’s Republic of China; e School of Rehabilitation, Sport and Psychology /Research Centre for Health, Exercise and Sports Science, AECC University College, Bournemouth, England; f Body-Brain-Mind Laboratory, School of Psychology, Shenzhen University, Shenzhen, People’s Republic of China

**Keywords:** Uncertainty stress, negative affect, subjective sleep quality, self-rated health

## Abstract

**Objectives:**

The COVID-19 crisis caused unparalleled uncertainty stress and health-related symptoms among Chinese residents. This study aimed to characterize stress status during the early stage of the pandemic and explore the inner mechanism between uncertainty stress and self-rated health.

**Setting/participants:**

A cross-sectional design was conducted online from February 7 to 14, 2020. A total of 2534 Chinese participants were surveyed.

**Main outcome measures:**

Uncertainty stress, negative affect, sleep quality, and health status were measured by self-report. A sequential mediation model using bootstrapping method was applied to test these relationships.

**Results:**

Age, place of residence, marital status, occupation, household annual income, infection, and quarantine status significantly correlated with uncertainty stress. Higher uncertainty stress was negatively related with self-rated health (r = −0.256, *p* < 0.01) and positively associated with higher negative emotions (r = 0.646, *p* < 0.01). The sequential mediation model found total indirect effect (β = −0.014, 95%C.I. = −0.017−0.010) and direct effect (β = −0.010, 95%C.I. = −0.015−0.005) were significant in the relationship between uncertainty stress and self-rated health with mediating by negative affect and subjective sleep quality.

**Conclusions:**

Findings provided evidence-based information for stakeholders designing and implementing intervention strategies by providing psychological consultation services and public education to manage uncertainty stress and minimize the damage of negative affect and poor sleep.

## Introduction

On the 30th of January, 2020, the World Health Organization (WHO) declared the outbreak of COVID-19, an identified novel coronavirus, which was first reported in Wuhan City, Hubei Province, a public health emergency of international concern (Alqahtani et al., 2021). Extensive severe public health policies have been implemented in response to prevent the global spreading of this virus, including avoidance of public contact and quarantines (Adhikari et al., 2020). It is worth noting that the COVID-19 pandemic and the protective measures during the first wave worsened the mental state of the general population and self-rated health (SRH) (Peters et al., 2020). SRH has been found to be one of the most powerful predictors of health, clinical outcomes, morbidity and mortality (Fayers & Sprangers, 2002; Goldman et al., 2004; Idler & Benyamini, 1997). While some people tended to think of ‘health’ as physical health, others may have used a frame of reference that included emotional or mental well-being. It has also been reported that the perceived negative impact of the COVID-19 crises on work and private life and mandatory short-time work significantly changes SRH (Tušl et al., 2021). However, these studies were unable to explain the extensive association between SRH and the mental dimension, and did not consider the synergy or antagonism among these psychological variables associated with COVID-19. Understanding the influencing mechanism of self-rated health during the COVID-19 pandemic might help the residents to maintain a general subjective sense of healthiness and provide supporting evidence for the design and implementation of interventions for pandemics ahead.

There were various factors that correlated with the public’s health during the COVID-19 pandemic. It had been reported that mental health problems were among the most detrimental factors associated with SRH during the crisis (Szwarcwald et al., 2021). Along with the previous studies, uncertainty stress had significant correlations with the mental health and a variety of stress factors (Wang et al., 2020; Wu et al., 2020) and had been identified as the greatest single psychological stressor for patients with a life-threatening illness (Koocher, 1985). COVID-19 has rapidly become a disease associated with unbridled uncertainty with its etiology and management (Koffman et al., 2020). When thrown upon such swift change in every aspect of life, people were overwhelmed with uncertainty. Given the sustained uncertainties, COVID-19 may persist and continue to impact health status. However, there was little literature to explore the mechanism by which uncertainty stress affected health during the COVID-19 pandemic.

According to stimulus-response theory, reception of a particular stimulus can be physiologically associated with the production of a particular reaction (Treisman, 1960). There might be a path where uncertainty (a stressor as external stimulus) causes perceived stress and stress responses which then act on negative affect (emotional reaction), poor sleep quality (behavioral reaction) and health status (physiological reaction or general health outcomes). Negative affect is a personality variable that involves the experience of negative emotions and poor self-concept (Watson & Clark, 1984). A number of studies observed negative affect during the COVID-19 restrictions (Lades et al., 2020; Li et al., 2020; Zacher & Rudolph, 2021). There might be an association, which was processed with uncertainty tolerance and emotion regulation strategies, between uncertainty and negative affect (Anderson et al., 2019; Carleton, 2016). Sleep problems had also become a major health concern in COVID-19. An online questionnaire survey that targeted Wuhan residents showed 30% of participants had insomnia symptoms starting from the lockdown (Fu et al., 2020). The negative affect including higher depression, anxiety and stress symptoms reduced sleep quality (Stanton et al., 2020). Further, insufficient sleep duration was significantly associated with worse self-rated health and more overall, as well as specific psychosomatic health complaints including headache and backache (Kosticova et al., 2019).

Based on the literature above, uncertainty stress and negative affect seemed to be the elements of cognitive vulnerability impacting sleep quality and in turn, health status (Kocevska et al., 2020). The current study sought to explore the mechanism affecting health status within the early outbreak of COVID-19, whether negative affect acted as a mediating factor between uncertainty stress and sleep quality, further to explore how mental factors work with each other in relation to self-rated health in a sample of general Chinese citizens.

## Materials and methods

### Study design and participants

This study utilized a cross-sectional correlational design. Our survey was developed on the platform named Wenjuanxing (https://www.wjx.cn/app/survey.aspx) and conducted online during the second week in February 2020. Twenty psychology students were enlisted and trained as research assistants. Snowball technique was applied through WeChat and other websites to recruit participants. More detailed information on participants recruitment can be found in another published paper (Wu et al., 2021). Our sample covered 30 provinces, municipalities, autonomous regions of China, and regions abroad. The study protocol was approved by the Ethics Committee of Shenzhen University. Based on the previous study, a total of 2534 participants with prior written consent took on average 15-minutes to complete the questionnaire in the survey. Only the responses to the questionnaires that followed the criteria of filling out in reasonable time, completeness and consistency were processed as valid.

### Measures

The survey questionnaire covered five categories: (a) demographics, (b) uncertainty stress, (c) negative affect, (d) subjective sleep quality (SSQ), and (e) self-rated health.

### Dependent variables

#### Self-rated health

Self-rated health (SRH) is a measure of a respondent’s subjective sense of health (Snead, 2007), which is commonly used to capture a general sense of health from the perspective of the participant. Methodologically, the SRH has been found to be both a reliable and valid measure of health status (Lundberg & Manderbacka, 1996). The participants had to rate their health on a 5-point scale with responding alternatives: ‘How would you rate your health status overall during the past month: very bad, bad, fair, good, or very good?’ Their answers yielded health status scores with a possible range from 1 to 5 as a higher score represents a better self-rated health status (Laaksonen et al., 2005).

### Independent variables

#### Demographic characteristics

The sociodemographic features were collected during the survey while specific experience with COVID-19, including infection and quarantine status, were also measured. The details can be found in the previously published work on this sample (Wu et al., 2021).

#### Uncertainty stress

The 10-item Uncertainty Stress Scale was designed by Yang et al and measured uncertainty stress in the social context (Wu et al., 2016; Yang, 2018). A 5-point Likert-type scale was applied to evaluate perception of stress which resulted from uncertain situations with each item score ranged from 1 (very little stressful) to 5 (extremely stressful). Item scores were summated to obtain a total score with the higher the score equals to the greater the uncertainty stress. The Uncertainty Stress Scale included the following ten situations, (1) Life is impalpable, and fate is unpredictable; (2) Feeling things are not going well; (3) Social values are chaotic, and I am experiencing confusion; (4) Unexpected things often happen in life; (5) The world is changing too fast and I cannot keep up; (6) I do not know how to reach my own goals; (7) Confused about the future; (8) Many people ignore the rules and I do not know what to do; (9) Inability to handle important changes in life; and (10) Feeling there are no rules and paths to follow. The Cronbach’s alpha coefficient for the Uncertainty Stress Scale was 0.925 in this study, indicating great reliability.

#### Negative affect

Negative affect (NA) was measured by the negative affect part of Positive and Negative Affectivity Scale (PANAS) (Watson et al., 1988). The PANAS consists of two 10-item scales assessing positive affect (active, alert, attentive, determined, enthusiastic, excited, inspired, interested, proud, and strong) and negative affect (distressed, afraid, ashamed, guilty, hostile, irritable, jittery, nervous, scared, and upset). Each item of PANAS was rated on a 5-point Likert-type scale (from not at all/ very little to extremely) resulting in the range for each scale (10 items on each) is from 10 to 50. The higher the score, the greater the negative affect. The Cronbach’s α coefficient for the Negative Affectivity Scale part was 0.926.

#### Subjective sleep quality

To assess SSQ, the participants were asked: ‘How would you rate your sleep quality overall in past month?’ The item was divided into four levels with a 4-point Likert scale. The responses were coded as 1 = very well, 2 = fairy well, 3 = fairly poorly, 4 = very poorly. The higher the score, the worse the sleep quality.

#### Data analysis

All survey data were entered into a Microsoft Excel database, and then imported into SPSS (version 22.0). Descriptive statistics and univariate analysis on uncertainty stress were conducted. Cronbach’s alpha coefficient and exploratory factor analysis were used to examine the reliability and validity of the Uncertainty Stress Scale and Negative Affectivity Scale. Pearson correlational analysis was applied to explore the relationships among uncertainty stress, negative affect, sleep quality, and health status. A sequential mediation analysis using the bootstrapping method was performed with PROCESS. With regard to the mediation model, the dependent variable was SRH, and US was the independent variable. The mediators were NA and SSQ, and the covariates were the significant demographic characteristics, infection, and quarantine status via univariate analysis. In the present study, the 95% CI of the direct effect and total indirect effects was obtained with 5000 bootstrap resamples. A significant indirect effect via mediators between dependent and independent variables was identified if the 95% CI does not contain zero.

## Results

A total of 2534 participants in the survey, of whom 2215 (87.4%) completed valid questionnaires. Among this general public sample, about half were aged 20 to 24 years old (50.2%), over two-thirds of the participants were female (67.2%) and urban residents (68.7%), 75.3% were unmarried and 59.2% were students. It was worth noting that 54 respondents were infected by COVID-19 (2.4%). A more detailed description of the characteristics was summarized in Table 1. The statistically significant differences in sociodemographic characteristics for both total uncertainty stress and self-rated health were for age, occupation, and household annual income (RMB). Their socially related ones’ quarantine status due to infection or suspected infection by COVID-19 were all significantly correlated with US and SRH.

**Table 1.** Uncertainty stress and self-rated health among different demographic characteristics.

Variables	*N*	%	US Mean (SD)	SRH Mean (SD)
**Age**			F = 8.517, *p* < 0.001**	F = 4.529, *p* = 0.001**
<20	411	18.6	26.82 (8.04)	4.23 (0.78)
20–24	1113	50.2	28.31 (8.31)	4.08 (0.79)
25–29	240	10.8	27.93 (9.39)	4.00 (0.87)
30–39	209	9.4	26.72 (9.06)	4.12 (0.82)
40+	242	10.9	25.10 (8.58)	4.20 (0.80)
**Gender**			t = 0.527, *p* = 0.468	t = 3.409, *p* = 0.001**
Male	726	32.8	27.68 (9.15)	4.20 (0.81)
Female	1489	67.2	27.40 (8.30)	4.08 (0.79)
**Place of residence**			t = 8.803, *p* = 0.003**	t = −0.155, *p* = 0.877
Urban	1522	68.7	27.12 (8.67)	4.12 (0.80)
Rural	693	31.3	28.29 (8.35)	4.12 (0.80)
**Ethnicity**			t = 1.745, *p* = 0.187	t = 2.31, *p* = 0.021*
Han	2155	97.3	27.45 (8.58)	4.13 (0.79)
Minority	60	2.7	28.93 (8.61)	3.88 (1.04)
**Marital status**			F = 8.377, *p* < 0.001**	F = 0.669, *p* = 0.512
Unmarried	1669	75.3	27.79 (8.30)	4.11 (0.79)
Married	517	23.3	26.32 (9.30)	4.15 (0.82)
Divorced/widowed	29	1.3	31.03 (9.45)	4.03 (1.18)
**Education**			F = 1.848, *p* = 0.136	F = 0.545, *p* = 0.652
Junior high school or less	196	8.8	26.94 (9.28)	4.18 (0.88)
High school	233	10.5	28.68 (9.55)	4.09 (0.82)
Junior college	263	11.9	27.32 (9.12)	4.12 (0.83)
College or higher	1523	68.8	27.41 (8.23)	4.11 (0.78)
**Occupation**			F = 3.427, *p* = 0.008**	F = 2.487, *p* = 0.042*
Public official/professionals	257	11.6	25.82 (9.57)	4.01 (0.85)
Enterprise personnel	238	10.7	27.68 (9.12)	4.13 (0.82)
Commerce/service/operations	215	9.7	28.30 (8.96)	4.06 (0.84)
Students	1311	59.2	27.73 (7.98)	4.16 (0.77)
Others	194	8.8	26.92 (9.74)	4.06 (0.88)
**Household annual income (RMB)**			F = 10.672, *p* < 0.001**	F = 4.05, *p* = 0.028*
Less than 20,000	475	21.4	28.15 (8.62)	3.033 (0.82),
20,000–60,000	832	37.6	27.94 (8.43)	4.10 (0.80)
60,000–100,000	516	23.3	27.83 (7.67)	4.14 (0.80)
More than 100,000	392	17.7	25.29 (8.58)	4.20 (0.77)
**Infected by COVID-19**			t = 5.044, *p* < 0.001**	t = −1.447, *p* = 0.148
Yes	54	2.4	33.28(8.68)	3.96 (0.82)
No	2161	96.5	27.34(8.53)	4.12 (0.80)
**Friends/colleagues/relatives quarantined due to COVID-19**			t = 4.604, *p* < 0.001**	t = −3.670, *p* < 0.001**
Yes	132	6.0	30.81(9.14)	3.87 (0.84)
No	2083	94.0	27.28(8.51)	4.13 (0.80)
**Neighborhood quarantined due to COVID-19**			t = 5.276, *p* < 0.001**	t = −3.166, *p* = 0.002**
Yes	278	12.6	30.01(8.46)	3.97 (0.81)
No	1937	87.4	27.13(8.54)	4.14 (0.80)

* < 0.05; ** < 0.01; Note: US: uncertainty stress; SRH: self-rated health.

The descriptive statistics and bivariate correlations were displayed in Table 2. The item score for self-rated health and SSQ were 4.12 (95% C.I. 4.08–4.15) and 3.01 (95% C.I. 2.97–3.04), respectively. The mean total score of uncertainty stress and negative affect were 27.49 (95% C.I. 27.13–27.86) and 23.47 (95% C.I. 23.12–23.83), respectively. The correlation between uncertainty stress and negative affect (r = 0.646, *p* < 0.001) was positive, as well as SSQ and self-rated health (r = 0.399, *p* < 0.001). Nevertheless, uncertainty stress (r = −0.227, *p* < 0.001) and negative affect (r = −0.226, *p* < 0.001) pessimistically related to SSQ.

**Table 2.** Inter-correlations and descriptive statistics of study variables.

	Variables					Total Score	
		1	2	3	4	M(SD)	95% C.I.
**1**	**US**	1.00				27.49 (8.58)	27.13–27.86
**2**	**NA**	0.646**	1.00			23.47 (8.26)	23.12-23.83
**3**	**SSQ**	−0.227**	−0.226**	1.00		3.01 (0.72)	2.97–3.04
**4**	**SRH**	−0.256**	−0.252**	0.399**	1.00	4.12 (0.80)	4.08−4.15

Note: US: uncertainty stress; NA: negative affect; SSQ: subjective sleep quality (1–4); SRH: self-rated health (1–5).

The findings from sequential mediation analysis using the bootstrapping method were presented in Tables 3 and 4. The total indirect effect (β = −0.014, 95%C.I. = −0.017−0.010) and direct effect (β = −0.010, 95%C.I. = −0.015−0.005) of uncertainty stress on health were significant respectively after adjusting for potential covariates including demographic characteristics. Regarding indirect effect, the sequential mediation effect from US to NA to SRH (β = −0.006, 95%C.I. = −0.010−0.003) and US to SSQ to SRH (β = −0.004, 95%C.I. = −0.006−0.003) were significant and further, the sequential mediation effect from US to NA to SSQ to SRH was also significant.

**Table 3.** The results from mediation analysis using a bootstrapping method for self-rated health.

Dependent variable	Independent variable^a^	Beta coefficient	*t*	*p*	R2	*p*
NA	*Constant*	24.078	10.95	<0.001**	0.46	<0.001**
	US	0.607	39.39	<0.001**		
SSQ	*Constant*	3.598	14.02	<0.001**	0.07	<0.001**
	NA	−0.013	−5.32	<0.001**		
	US	−0.011	−4.84	<0.001**		
SRH	*Constant*	3.375	12.26	<0.001**	0.21	<0.001**
	SSQ	0.387	17.67	<0.001**		
	NA	−0.010	−4.14	<0.001**		
	US	−0.010	−4.17	<0.001**		

^a^ All models were adjusted for infection, quarantine status, and demographic characteristics, such as age, ethnicity, residence, marriage. Bootstrapping = 5000; ** < 0.01; Note: US: uncertainty stress; NA: negative affect; SSQ: subjective sleep quality; SRH: self-rated health.

**Table 4.** Indirect effect of uncertainty stress on self-rated health via negative affect and subjective sleep quality.

Path	Coefficient	95% confidence interval	
		Boot lower limit	Boot upper limit
US→NA→SRH	−0.006	−0.010	−0.003
US→SSQ→SRH	−0.004	−0.006	−0.003
US→NA→SSQ→SRH	−0.003	−0.004	−0.002
Total indirect effect	−0.014	−0.017	−0.010
Direct effect	−0.010	−0.015	−0.005

Note: US: uncertainty stress; NA: negative affect; SSQ: subjective sleep quality; SRH: self-rated health.

## Discussion

The COVID-19 pandemic has brought unparalleled uncertainty stress to individuals and society since the early stage of the outbreak (Wang et al., 2021). Even after the massive vaccination process, the uncertainty stress of newly emerged variants or social-economical turbulence will continue perpetually. The current study reported the self-rated health status of Chinese residents during the third week of lockdown in Wuhan and explores the potential risk factors for influencing subjective health from the perspective of uncertainty. We discovered that young men, rural residents, divorced/widowed people, commerce/service/operations and those with lower household annual income had a higher total score of uncertainty stress, suggesting they were more vulnerable to the risk of experiencing extreme stress, partially in accordance with a previous study (Wang et al., 2021). Generally, the self-rated health was considered well at the beginning of the pandemic. However, ethnic minority, low household income and related ones’ quarantine status might have an effect on health. Other studies support the view (Glowacz & Schmits, 2020; Shah et al., 2021), that the government should pay more attention to these populations for their mental health issues. COVID-19 infected participants and those whose friends, relatives, colleagues, or neighbors were quarantined due to infection or suspected infection by COVID-19 had a significantly higher uncertainty stress score than their counterparts. This was in line with the results from another study showing close contact with patients with COVID-19 was associated with mental health status (Hossain et al., 2020). When confronted with direct or indirect COVID-19 infection type of uncertainty stress, individuals tend to struggle with new information and reluctantly to adopt preventative behaviors. A previous study showed that uncertainty stress was positively associated with disease fear, and negatively associated with prevention behaviors (Peng et al., 2021). Controlling uncertainty stress is an important aspect in the prevention of COVID-19 infections.

Our result from the sequential mediation models suggested that uncertainty stress during the early stage of COVID-19 outbreak might correlate with the general public’s self-rated health. The Illness Uncertainty Theory explains how patients cognitively process an illness-related stimulus, as well as how they structure the meaning of such an event, and proposes that high uncertainty is associated with diminished capacity to process new information, predict outcomes, and adapt to the illness (Mishel, 1990). The COVID-19 pandemic, however, is changing – or has already changed – our collective evaluation of uncertainty because there is no reference case for the COVID-19 crisis in living memory. A recent study revealed a higher level of uncertainty stress is positively associated with mental disorder (Wu et al., 2020). It was consistent with an online survey showing poor self-rated health status significantly associated with a greater psychological impact of the outbreak and higher levels of stress (Wang et al., 2020). The plausible mechanism was uncertainty about COVID-19 would lead to cognitive confusion, exhaust an individual’s energy, diminish perceived control, and divert attention from routine healthy behaviors which might associate with perceived health (Peng et al., 2021; Wu et al., 2021).

Further, our sequential mediation analysis found there could be several indirect pathways between uncertainty stress and self-rated health. First, a positive association was found between uncertainty stress and negative affect agreeing with the previous studies (Araujo et al., 2020; Caffo et al., 2020). There could be several mediators between uncertainty and affect, including context and individual differences such as uncertainty tolerance, as well as emotion regulation strategies (Anderson et al., 2019). Furthermore, the potential mechanism that intolerance of uncertainty (IU) about COVID-19 was significantly and positively correlated with negative emotions would play an important part (Dai et al., 2021). In addition, a research study in the UK examined the general population was struggling with uncertainty during the first wave of the COVID-19 pandemic more so than normal (Rettie & Daniels, 2021).

Second, consistent with prior sleep research, it has been shown in the current study that uncertainty stress triggered negative impact on sleep quality in people who faced sudden events. The probable reason was that traumatic events such as those caused by COVID-19 outbreak could produce psychological distress and anxiety symptoms which negatively impacted sleep quality (Brooks et al., 2020). Compared to the pre-lockdown period, there was a shift to a later bedtime and waking time, with a reduction in nighttime sleep and an increase in day-time napping because of uncertainty associated with the pandemic (Gupta et al., 2020). In turn, poor sleep quality led to poor health status.

Third, we also found negative affect was a significant influencing mediator between uncertainty and two dependent variables as SSQ and self-rated health. There were negative changes in physical activity and sleep associated with higher depression, anxiety, and stress symptoms in Australia during COVID-19 (Stanton et al., 2020). Among senile populations, higher negative affect predicts worse self-rated health (Segerstrom, 2014). Negative affect has also been related to health status by sleep quality. An integrative bibliographical review suggested sleep exercised a direct effect on immunity maintenance and immunological response (Silva et al., 2020). Circadian rhythm alterations, associated with the psychological problems imposed by the COVID-19 pandemic compromise the quality of sleep and, for this reason, the immune system (Silva et al., 2020).

## Limitations

The study has some limitations. In the first place, the majority of participants are young college students, female, and urban residents, which suggest sample bias and may not represent the generally pooled Chinese population. Second, we could not infer causal conclusion because of cross-sectional design. We tested multiple indirect effects from uncertainty stress to health status by mediation analysis which capitalized on the large sample. Finally, SSQ was assessed by a single-item without objective measures. Therefore, answers may reflect not only sleep quality but also satisfaction and perception of sleep conditions. Future studies should use additional objective measures for sleep to verify these associations (Makizako et al., 2021).

## Conclusions

Our study revealed that Chinese residents’ uncertainty stress is directly associated with poor health, though, also indirectly related to the mediating role of negative affect and SSQ. The study might provide evidence-based information to the governments and policymakers to conceive valid health intervention strategies by providing psychological consultation services and public education for reducing the uncertainty stress, adjusting negative affect, and improving sleep quality and health status during the following stage of the COVID-19 era, especially the vulnerable population could receive greater attention.

## Data Availability

The datasets used and analyzed during the current study are available from the corresponding author on reasonable request.
